# Medical Opinion and Movement

**Published:** 1910-06-11

**Authors:** 


### Medical Opinion and Movement.

### Massage of the Spleen in Latent Malaria.

Dr. Pennato has already recorded three cases of malaria latent for several years in which energetic and prolonged massage of the spleen was sufficient to produce typical attacks of fever. Dr. Fabiani now records a similar case. The patient was a man aged 53, who had recently contracted a malarial infection. The fever appeared to have definitely disappeared under treatment with quinine. On examination the spleen was just palpable at its lower border, and was enlarged in volume, measuring 24 centimetres longitudinally and 10 centimetres transversely. The temperature varied between 36.3° C. in the morning and 37.3° in the evening. Repeated and careful examination of the blood for the parasite failed to reveal its presence. But after palpation and compression of the lower margin of the spleen for some minutes the patient was seized about 30 hours later with an attack of fever and profuse sweating, the temperature reaching 39.2° C. Examination of the blood 48 hours after massage of the spleen showed the presence of intracorpuscular forms of the parasite occupying almost the whole of the corpuscles. The febrile attacks recurred daily to the number of five, following the classic quotidian type, the maximum temperature being 40.3° C., but eventually they yielded to treatment with quinine.

### Injections of Hydrogen Peroxide.

Some years ago Drs. Cionini and Marcantoni, of Pisa, advocated injections of hydrogen peroxide solution for cases of tuberculous peritonitis and pleurisy, and published details of four cases of peritonitis and one case of pleurisy in which the treatment had been carried out successfully. Since then they have persistently employed this method of treatment. Altogether they have now carried out the treatment in 35 cases, including 29 cases of tuberculous peritonitis and six cases of pleurisy, in most of which ordinary medical treatment as well as thoracentesis, and in three cases laparotomy, had previously failed to produce any improvement in the condition. In all these cases a single injection of hydrogen-peroxide solution was sufficient to effect a cure of the disease. In some cases in which the peritoneum and pleura were affected simultaneously, treatment of the peritoneal cavity was followed by an absorption of the pleuritic effusion also. The condition generally cleared up in the course of one to seven weeks after the injection. Care must be taken to ascertain the acid employed to preserve the peroxide of hydrogen. Phosphoric or sulphuric acid are innocuous, but nitric or hydrochloric acid must be avoided. Having punctured the peritoneum, 1½ litre of a physiological solution of sodium chloride heated to 40° C. are injected, 75 cubic centimetres of hydrogen peroxide being added at the time, so as to make a 5-per-cent. solution. In children about 400 cubic centimetres of the solution should be injected. For the pleura the authors have not exceeded 1 litre. After injection the position of the patient should be changed, so as to bring the solution in contact with all parts of the pleura or peritoneum, and at the end of about six to eight minutes the fluid is evacuated. The patient should be kept absolutely quiet for several hours. A few hours after the injection there is usually a considerable rise of temperature, which gradually subsides in the course of a few days. There is often also a great acceleration of the pulse and general signs of collapse, but these subside in the course of a few hours. Tuberculous masses tend also to disappear after the injections. In the cases of pleurisy the post-operative signs are much less marked.

### Local Injections of Sodium Salicylate.

The treatment of rheumatic and gonorrhœal joints by local injections of sodium salicylate was first suggested by Dr. Bouchard, but has not hitherto received much support. Dr. R. Rosenthal has, however, made a special study of the question, and advocates a more extended use of the method. A sterilised solution of 5-per-cent. sodium salicylate is used, and 1 or 2 cubic centimetres are injected with a Pravaz syringe into the periarticular connective tissue. The injection produces a burning sensation, which, however, passes off in the course of 15 or 20 minutes. This may be obviated by adding 1 per cent. of cocaine to the solution. In acute cases one or two injections suffice, but in the more chronic affections as many as 30 injections may be made, and they may be given every day or every other day at different points around the joint. The treatment is especially indicated in mon-arthritic cases, or in cases where sodium salicylate is not well tolerated by the mouth. It has the advantage of acting locally and directly on the joint without saturating the system with large doses of the drug, only a small portion of which can ever reach the affected part. On the other hand, in general acute rheumatic fever local treatment would be contra-indicated, except perhaps in cases in which absolute intolerance of the drug is shown. For such cases local injections might be made around the affected joints.

### Urea in the Cerebro-spinal Fluid.

According to Dr. J. Froment, estimation of the urea content of the cerebro-spinal fluid by means of lumbar puncture may furnish important clinical evidence in the diagnosis of cerebral conditions of uræmic origin. The cerebral symptoms, be they convulsive or paralytic, may present nothing distinctive in themselves, and the diagnosis must often rest upon other pathological evidence on the part of the urinary or cardio-vascular systems. Examination of the cerebro-spinal fluid, too, in regard to its molecular concentration, percentage of chlorides or toxicity does not disclose any typical changes in these conditions, but estimation of the percentage of urea appears to yield more definite indications. In normal conditions the amount of urea in the cerebro-spinal fluid is practically negligible, amounting to about 15 centigrammes and never reaching 1 gramme per 1,000. On the other hand, in uræmia with cerebral symptoms it often amounts to 1.5 gramme, and even 4.5 grammes, per 1,000. The author gives details of two cases in which a diagnosis founded on the urea content of the cerebro-spinal fluid was confirmed at the autopsy. The first case was that of a woman who was admitted to hospital in a condition of stupor, and soon developed coma. Jaundice followed, accompanied by a right hemiplegia. The urine contained a large amount of albumen. The cerebro-spinal fluid was quite clear and did not show a trace of urea. At the autopsy the kidneys were small, the aorta markedly atheromatous, and the brain showed foci of softening scattered throughout. The second case was a man aged 52, who had had epileptic fits three months previously and well-marked albuminuria. He was in a condition of stupor, quite indifferent to his surroundings, with respirations much accelerated; 30 c.c. of cerebro-spinal fluid were withdrawn by lumbar puncture, yielding 4.5 grammes of urea per 1,000. The patient died after a convulsive fit, and the autopsy showed a chronic nephritis but no cerebral lesion. In regard to prognosis the author thinks that if the quantity of urea exceeds 4 grammes per 1,000 a fatal issue is indicated, whereas 1 or 2 grammes per 1,000 point to a more favourable prognosis.

### Nitrous Oxide Gas and Osteo-arthritis.

Messrs. Relph and Rood contribute to the *British Medical Journal* the account of a difficult case of nitrous oxide anæsthesia which is almost the exact duplicate of one recorded by Dr. Hewitt in his text-book of anæsthetics. The patient was a young man suffering from multiple osteo-arthritris, which had affected his temporo-mandibular joints to such an extent that the teeth could only be separated about a third of an inch. It so happened that the teeth were in such a bad state that it was thought advisable to remove most of them, or at least of the stumps which alone remained where teeth should have been. Nasal respiration was quite free. Gas was given by the nasal method, and after one root had been removed it was noticed that cyanosis was supervening, notwithstanding that some air was entering. The anæsthetic was discontinued, but respiration ceased entirely. A Mason's gag was put in and opened, the pharynx sponged clear of mucus, and the tongue pulled forward, but without avail. The man was then laid on the floor and artificial respiration was begun, but no air entered the chest owing to laryngeal obstruction. Tracheotomy was then at once performed, and oxygen delivered through the wound; after a few artificial breaths natural respiration recommenced and the patient was soon all right. Apparently congestion and swelling of the tissues about the glottis was the cause of the obstruction, as in Dr. Hewitt's case. The moral is that instruments for tracheotomy and an oxygen cylinder should always be at hand whenever even the simplest anæsthetic is to be administered to anyone whose air-way is in the least obstructed or liable to become so.

### The Causation of Goitre.

The experiments and researches of Captain McCarrison in Gilgit, India, into the causes of endemic goître there, and his conclusions that the prime agent is some micro-organism present in the water supplies, were summarised in these pages more than a year ago. Dr. Colquhoun, impressed by the frequency of goître in New Zealand, especially in certain districts, has collected an extensive mass of information by circularising members of the profession in different districts. He sums up the results thus obtained in the following statements, each of which is ably supported by a large mass of evidence. The female sex about the age of puberty and for some years afterwards is especially predisposed to enlargements of the thyroid gland in certain parts of New Zealand. The exciting agency is to be looked for in the drinking waters; it is something which is common to rain-water, artesian wells, and natural hot springs. The only common factors in these waters are the radio-active substances or their derivatives, but the physical evidence is not as yet conclusive on this point. It may also be that different agencies are at work in different districts, though this is unlikely: possibly a parasite is the cause in Gilgit, but it is something else in New Zealand.

### Hypopituitarism.

Definite constitutional disturbances which are regarded as manifestations of hypopituitarism have been observed after partial anterior lobe removals in animals kept under observation for long periods. The most striking is a state of adiposity with hypoplasia of the generative organs in adults or with persistence of sexual infantilism when the animals operated on have been sexually immature. Polyuria, glycosuria, œdemas of the skin and hypotrichosis, subnormal body temperature, and psychic disturbances are more or less frequent accompaniments. All these symptoms have been recorded with adiposity and sexual infantilism in man associated with pituitary tumours; and therefore are due presumably to hypophyseal deficiency. Animals in a condition of hypopituitarism have a lowered resistance to infection and disease; and intercurrent disease may precipitate the onset of cachexia hypophyseopriva. It has been possible by glandular transplantations, or by injections of anterior lobe emulsion, definitely to prolong the life of animals after total hypophysectomy, and also to tide over threatened cachexia hypophyseopriva in animals retaining anterior lobe fragments which temporarily may be physiologically insufficient.

### Tubal Gestation Extraordinary.

A most unusual, indeed probably unique, case of tubal gestation is reported from Buenos Aires by Mr. Morris in the *Lancet*. A Hungarian woman was admitted to the British Hospital in that city complaining of an abdominal tumour associated with thirteen months' amenorrhœa. About six months after the cessation of menstruation quickening was perceived, and the movements were felt for about three months. After this had ceased the abdomen was said to have diminished in size, and the breasts, which had been secreting, became once more inactive. On bimanual examination the uterus was quite clearly detected separate from the abdominal tumour, which approached to within six centimetres of the xiphisternum. At the laparotomy an enormously distended but unruptured Fallopian tube was found, containing a complete gestation sac and a dead fœtus of 5¾ lb. weight. There was some desquamation, but no maceration. The tube was densely adherent to many of the surrounding structures, especially to the back of the uterus and to the omentum. The walls of the tube were, on the average, $\frac{1}{16}$ of an inch thick, and the placenta was situated in the posterior wall. In recording this extraordinary case the author remarks that facilities for consulting the literature are not at his disposal; but if such a case has ever been encountered before it must be one of the curiosities of obstetrics.

### The Functions of the Pituitary Gland.

In the *Bulletin of the Johns Hopkins Hospital* appears a very lengthy and thorough account of a most exhaustive research by Drs. Crowe, Cushing, and Homans into the functions of the pituitary body, and the effects of removing the whole or parts of it. Previous investigators have not been in agreement as to the physiological essentiality of the hypophysis cerebri, due largely to the difficulty of removing the structure without damaging also the brain and other important parts. The most elaborate precautions were taken to exclude all such sources of error; and the authors agree with Paulesco on this point, holding that a state of apituitarism due to complete removal leads inevitably to death, with a peculiar and characteristic train of symptoms which they term cachexia hypophyseopriva. But even in adult animals death need not occur so rapidly as Paulesco thought; and puppies may even remain apparently normal for at least three weeks before the terminal phenomena appear. The same symptoms, at about the same intervals, follow the removal of the whole of the anterior pars alone, even when the posterior lobe is left intact. On the other hand, removal of the posterior lobe (except a small part of the pars intermedia) not only leads to none of the manifestations of cachexia hypophyseopriva, but does not appear to affect the physiological balance of the animal in any symptomatic way, unless convulsions and excessive sexual activity—which have been observed in a few cases—can possibly be ascribed to this operation.

### Agglutination Test for Actinomycosis.

According to M. F. Widal, who has recently made a communication to the Paris Academy of Medicine on the subject of the sero-diagnosis of actinomycosis, the disease is much more common than is generally supposed. It is of paramount importance to diagnose it early, in order to institute treatment by iodide of potassium, which when applied in the early stages is an effective remedy. When the face is the site of the disease, diagnosis is comparatively simple, but such is not the case when the affection is visceral or deep-lying. The author has for this reason attempted to discover a humoral reaction whereby diagnosis will be simplified. His first experiments consisted in attempting to cause agglutination of the mycelium by bringing it into contact with serum obtained from individuals suffering from the disease. The results were, however, negative. But on mixing the same serum with spores of sporotrychum a very pronounced agglutination was noticed with dilutions of 1 in 50 and 1 in 150. Six to twelve week old cultures on gelose to which glucose or maltose has been added up to 4 per cent., and which can be kept after sterilisation with formaldehyde vapour, should be used when applying the test. The author cited a case in which a doubtful diagnosis of visceral actinomycosis had been confirmed by this spore agglutination test, and which was rapidly cured after the administration of potassium iodide.

### Retro-Peritoneal Hernia.

In the May number of the *Annals of Surgery* A. C. Matthews has collected all the known cases of retro-peritoneal hernias near the cæcum, and has added one of his own. There are eight cases into the sub-cæcal fossa, of which three recovered; six cases into the ileo-appendicular fossa, of which two recovered, three died from the lesion, while one was discovered at an autopsy, not having given rise to any symptoms. There are no features distinctive of the lesion, and the symptoms are those of acute or sub-acute intestinal obstruction. The condition should be borne in mind when dealing with obscure abdominal cases.

### Injury as a Cause of Cancer.

In the same journal Charles Phelps reviews the opinions of over 300 authorities on the relation of trauma to cancer—a subject which has become important through certain law proceedings. The opinions fall under three heads: those who deny that any form of trauma is a cause of cancer, those who admit chronic persistent irritation or inflammation, and those who believe that single and transient injuries may be a cause of cancer. The second is the largest class, the third the smallest. The opinions in this last class are for the most part half-hearted, and when dogmatically stated are founded upon impressions only, and not on any scientific observations. If cases be excluded where the tumour has appeared so soon or so long after as to have little chance of possessing any causal relation, and also cases where the blow has called attention to an already existent tumour, the number of cases is surprisingly small where any history of injury is obtained. Phelps has found records of only seven of these, in addition to nine cases of so-called "acute traumatic malignancy," in the literature of the last 60 years. Sarcomata are not considered in this review; which is otherwise a very full and exhaustive paper on an interesting subject.

